# Impact of the Practice of Playing Video Games on Technical Skills Development in Preclinical Dental Education: Preliminary Cross-Sectional Observational Study

**DOI:** 10.2196/80082

**Published:** 2025-12-03

**Authors:** Roselyne Clouet, Thomas Remaud, Camille Boeffard, Samuel Serisier, Alexis Gaudin, Tony Prud'homme

**Affiliations:** 1 Service d’Odontologie (UIC11) Centre hospitalier universitaire de Nantes Nantes Université Nantes France; 2 Regenerative Medicine and Skeleton (UMR 1229) Centre hospitalier universitaire de Nantes (INSERM) Nantes Université Nantes France

**Keywords:** video games, dental education, surgical education, simulation, preclinical training

## Abstract

**Background:**

Video games are becoming increasingly accessible and occupy a prominent position among students’ leisure activities. Recent studies have demonstrated that engagement with video games can facilitate the development of specific abilities, such as visuospatial skills and hand-eye coordination. Thus, it seemed relevant to investigate whether the practice of playing video games could enhance the technical capabilities of novice dental students.

**Objective:**

The objective of this study was to ascertain whether dental students who identified themselves as video game players exhibited differences in fundamental technical skills in comparison to students who identified as nonplayers. This preliminary study aimed to validate the methodology and measurement tools for a subsequent prospective study.

**Methods:**

Second-year dental students who were novices in the field of preclinical dental practice were divided into 2 groups: one designated nonplayers and the other players. The visual, motor, and cognitive coordination of the students was assessed using 3 different tests. The initial assessment focused on evaluating spatial ability, while the subsequent assessments addressed arm-hand coordination and the velocity of execution. The study data were collected in September 2021.

**Results:**

This study included 92 second-year dental students (mean age 19.49, SD 0.8 years). Thirty-seven students were placed in the player group (40%), while 55 were placed in the nonplayer group (60%). The findings showed no statistically significant differences between the two groups when the 3 distinct tests were administered. The nonplayer group made fewer mistakes on the test evaluating spatial ability (*P*=.051) and achieved a higher score on the modified Precision Manual Dexterity Test, which evaluates arm-hand coordination, compared to the player group (*P*=.17), but without statistical significance. The nonplayer group took more time to perform the pulpotomy during the third test, which evaluated execution speed, compared to the player group, again without significance (*P*=.87).

**Conclusions:**

This study did not demonstrate significant differences between player and nonplayer dental students who participated in the study with regard to their fundamental technical abilities in a preclinical training environment. Nevertheless, it validated the feasibility of a methodology for a future longitudinal study to concentrate on the evolution of acquiring technical skills during preclinical training in these two populations. Consequently, further investigation is warranted to determine the potential impact of video games on the acquisition of surgical skills, including in dentistry.

## Introduction

Since the inception of the video game industry in the 1970s, the significance of these games in the lives of users and players has increased exponentially. Video games have become a major component of popular culture, with their use becoming pervasive from a young age and subsequently becoming an integral part of people’s lives. It is evident that video games have the capacity to exert a beneficial influence on society [1]. In 2022, players constituted almost 39% of the global population, and in countries with a high or very high Human Development Index, up to 71% of the population plays at least occasionally [2]. Consequently, players can be categorized according to their distinct gaming habits.

Despite the remarkable capacity of humans to acquire novel skills and adapt their behavior in response to experience, performance enhancements are frequently constrained by the parameters of the training environment [3]. Nevertheless, research conducted over the past decade has demonstrated that training involving action video games has the potential to yield learning that transfers beyond the immediate training context [3]. A significant number of psychological and educational studies have been conducted on the psychomotor effects that may be observed in individuals engaging in this practice, particularly among those rely on visual, motor, and cognitive coordination in their gaming activities.

A number of earlier studies reported that participation in video gaming was associated with enhancements in psychomotor functioning and other competencies. Indeed, players have been shown to outperform nonplayers in terms of the precision of their arm-hand movements and the efficiency with which they complete tasks [4,5]. Players have also been shown to track faster-moving objects, detect changes in visual short-term memory more efficiently, switch tasks more rapidly, and carry out mental rotation tasks more effectively than nonplayers [6]. Furthermore, players appear to exhibit increased perceptual reaction speeds without compromising performance accuracy [7].

However, it should not be forgotten that video games may also exert negative effects. The problems associated with them are mainly related to excessive use, including the potential for addiction, aggression resulting from violent games, and problems linked to a more sedentary lifestyle (obesity, musculoskeletal disorders, eye strain) [8,9].

On the other hand, students are increasingly fond of learning through games. Recent studies highlight the growing role of serious games as effective educational tools in dental education, enhancing engagement and learning outcomes. Studies have shown that gamified applications, such as ESKILLD for laser dentistry, SimOL for oral lesion diagnosis, and virtual environments like the Gamified Virtual Dental Clinic, significantly improve students’ satisfaction and knowledge acquisition. Although learning outcomes are often comparable to traditional teaching, serious games consistently promote higher motivation and positive learning experiences among dental students [10-13]. It is therefore relevant for dental educators to embrace these approaches in order to adapt to a student population whose abilities may be influenced by video games, but who are also receptive to and more interested in teaching methods that use serious games.

It is therefore pertinent to consider whether the extensive use of video games in medical practice exerts any influence on clinical competencies [14]. A number of studies have been conducted in order to observe the possible interactions between surgical skills and video gaming practice, with a particular focus on laparoscopic surgery. The results of these studies demonstrated a high degree of correlation with those from other fields, as video game skill correlated with higher laparoscopic surgical skills [15]. Indeed, prior experience with video games has been demonstrated to enhance baseline performance in laparoscopic simulator skills [14,16]. Nevertheless, it is evident that there exists a methodological heterogeneity amongst the various trials that have examined the impact of video games on surgical skills among medical students [17]. The conclusions are varied and occasionally contradictory. Consequently, the literature does not permit conclusions to be drawn on the consequences of a particular video game practice on the learning of technical skills in surgery. There is a paucity of evidence to support the hypothesis that the use of video games enhances surgical simulation performance [18]. Nevertheless, it appears that medical students hold a favorable opinion of the use of video games in a medical teaching context [19].

To date, there have been no studies examining video game use patterns among dental students and correlating them with preclinical technical performance using standardized testing. Preclinical training constitutes an indispensable element of dental education, with the objective of cultivating students’ manual dexterity and fundamental technical skills, which are essential for the successful execution of dental procedures. Indeed, it is imperative that they are able to comprehend the diverse anatomical configurations of teeth as depicted in medical imagery. Furthermore, concomitant development of arm-hand and visuospatial coordination is critical, particularly in the context of dental care using indirect vision [20].

In view of the growing literature pertaining to the contribution of video games in the medical field, we hypothesized that playing such games could influence preclinical learning in dentistry. In this pilot study, we included a wide variety of game genres. Rather than restricting inclusion to traditional action games (first-person shooter or role-playing games), we also included sports games, strategy games, racing games, platform games, and even smartphone games. The objective of this work was to ascertain whether the practice of playing video games could enhance the technical capabilities of dental novice students, thereby directly impacting their competencies during the preclinical training phase. Furthermore, given the absence of a preexisting standardized methodology, it was necessary to adapt evaluation tools for use in the dental field. A significant part of this study was therefore also dedicated to applying specifically designed or adapted psychomotor tests to assess skills that are directly relevant to dental education.

## Methods

### Study Design

This was an observational, descriptive, single-center, controlled, cross-sectional, and single-blinded preliminary study.

### Ethical Considerations

The research was approved by the Ethics, Deontology and Scientific Integrity Committee of Nantes University (IORG0011023). The regulatory qualification for noninterventional research was submitted to and validated by the committee (reference number 26062023-1). The recruitment of participants was voluntary, with written consent obtained. Data were anonymized. No compensation was provided to participants.

### Inclusion and Exclusion Criteria

Included students were in their second year of study in dentistry and were the legal age of majority. This ensured that participants were novices in preclinical training, minimizing the effect of skill improvement that appears with time. At the Faculty of Dental Surgery of Nantes, no hands-on practical training occurs in the first year. Therefore, we aimed to assess the students’ baseline technical performance.

The exclusion criteria were as follows: students who were repeating their second year of dental studies, students who were younger than 18 years, and students with medical conditions that could impair their ability to complete a practical training course under the same conditions as the other participants.

### Sample Design

The 2 investigators made an oral presentation on the study’s organization and objectives.

After the inclusion criteria were applied, the participants were invited to complete a questionnaire to determine their allocation to either the player or nonplayer group. Participants who indicated that they engaged in play “several times a day,” “daily,” “regularly” and “occasionally” were categorized as “players.” In contrast, those who played “rarely” or “never” were designated as nonplayers. Two extreme subcategories were also defined for participants, including those who indicated that they engaged in play “several times a day,” who were categorized as extreme players, and those who “never” played, who were designated as extreme nonplayers. Participants were coded anonymously to ensure that the investigator remained blinded.

### Outcomes

The primary outcome of this study was the influence of video games on the preclinical abilities of novice dental students. Comparisons between players and nonplayers were conducted for three skill domains that are particularly relevant in preclinical training: (1) 3D spatial representation, (2) accuracy of technical gestures, and (3) speed of execution. The secondary outcome was to compare these same domains between the extreme players and extreme nonplayers.

### Procedure and Outcome Measurements

**Figure 1:**
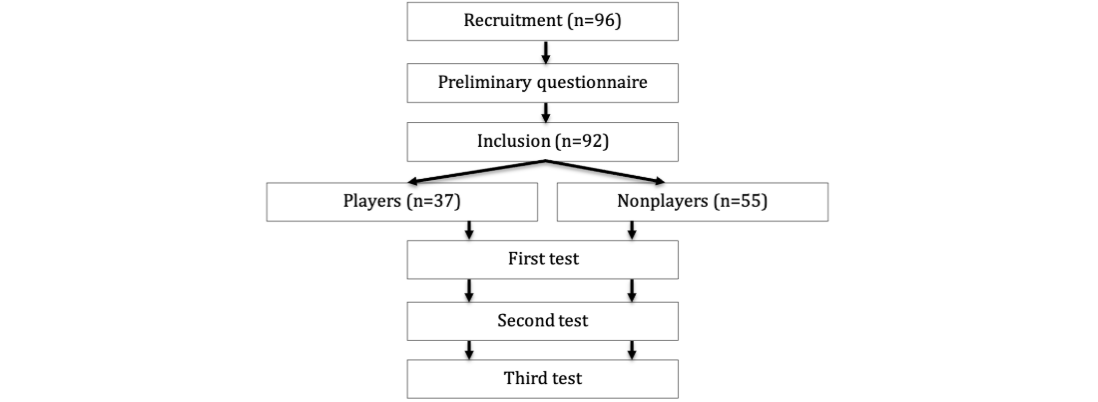
Flowchart of the study procedure.

Following the recruitment stage, participants completed a preliminary questionnaire to verify their eligibility and to allocate them to the appropriate groups. Subsequently, all participants were evaluated according to 3 distinct tests, corresponding to the 3 abilities mentioned above (Figure 1).

**Figure 2:**
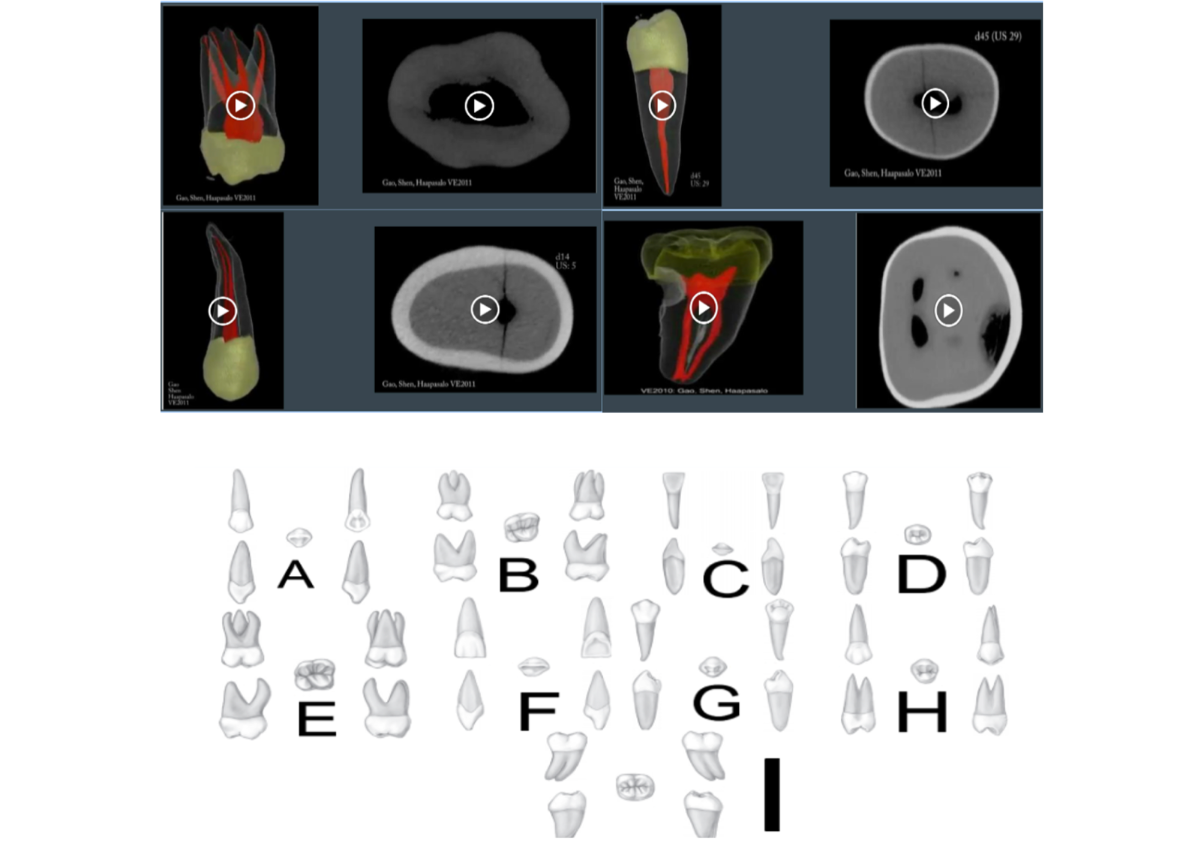
Presentation of the documents used in the first test. Top: participants associate videos of axial and sagittal sections of micro–computed tomography scans and 3D scanned models. Bottom: standardized anatomical sheets representing the 5 faces of the tooth.

The first evaluation comprised a task in which participants were required to match 3D and 2D images of the same tooth. This task served to assess the participants’ capacity to recognize an object from diverse perspectives and to evaluate their visuospatial abilities. Participants were asked to associate videos of axial and sagittal sections of a micro–computed tomography scan and 3D scanned models with the corresponding standardized anatomical board representing the 5 faces of the tooth (occlusal, mesial, distal, vestibular, and palatal/lingual; Figure 2). For each tooth, the participants received a point if they failed to correctly associate the projected 3D video representation with the correct 2D representation. The task consisted of accurately identifying 9 teeth, resulting in 9 assessments per participant. As one point was assigned for each error, higher scores indicated poorer performance.

**Figure 3:**
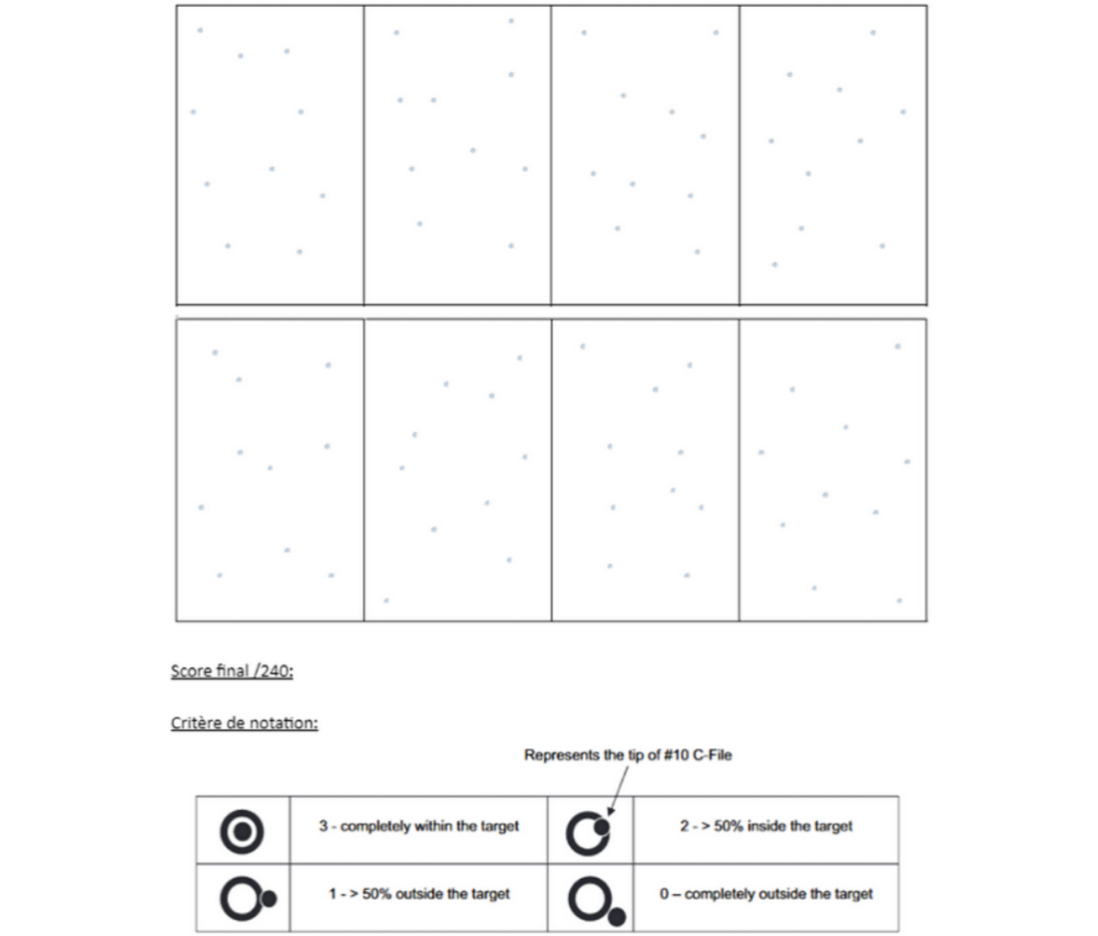
Illustration of the second test (modified Precision Manual Dexterity Test).

The second test assessed gesture precision and hand-eye coordination. We used a modified version of the Precision Manual Dexterity Test (PMD Test) as described by Bowers et al [21]. The test involved performing the most precise possible penetrations of 80 targets printed on a sheet of paper stretched over a soft support, using a handheld 10/100 steel endodontic file. Participants used their right hand to perform the task on the 4 rectangles located on the right side of the sheet (40 targets) and their left hand for the 4 rectangles on the left side (40 targets) (Figure 3). The test sheet was divided into 8 target zones, each containing 10 randomly arranged targets with an enlarged diameter of 1 mm, based on the original PMD Test, which was performed under optical magnification (×8 and ×2.5). Scoring was as follows: 3 points were awarded when the penetration point was entirely within the target, 2 points when more than 50% but less than 100% of the point was within the target, 1 point when less than 50% was within the target, and zero when the point was completely outside the target. Each sheet was visually compared to a prepunched reference sheet under backlighting for scoring. The maximum total score for each participant was 240 points.

**Figure 4:**
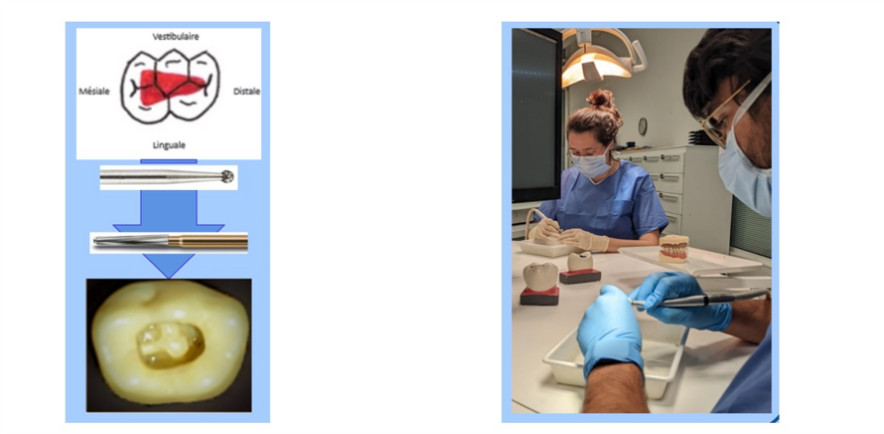
Illustration of the third test.

For the third test, the participants were required to undertake a complete pulpotomy procedure on a temporary mandibular molar resin model. The objective of this evaluation was to ascertain the efficiency with which a technical act was executed. In order to simulate clinical conditions and ensure maximum realism, a resin model of a right temporary second mandibular molar with a pulp chamber (Frasaco GmbH) was used (Figure 4). All participants received standardized written and visual instructions. The investigator demonstrated the procedure before the participants to clarify the success criterion. The success criterion was defined as the complete absence of any overhang above the chamber. Participants activated a stopwatch at the start of the procedure and stopped it upon reaching the chamber and notified the investigators, after which they were asked to remove any remaining overhangs. They stopped the stopwatch whenever they believed the success criterion had been met and requested an assessment from the investigators. The investigators then visually inspected the chamber opening to determine whether it met the quality requirement. If not, participants were instructed to continue; if acceptable, the procedure was deemed complete. The time (in seconds) required by the participants to correctly perform this pulpotomy by gaining access to the pulp chamber without producing an overhang was measured.

The comprehensibility and ease of use of the 3 tests were assessed by the 3 evaluators present during the sessions.

### Statistical Methods

Quantitative variables were described in terms of means and SDs. The collected data were then subjected to comparative analysis between the player and nonplayer groups. The parametric statistical test used in this study was the 2-tailed Student *t* test for independent samples. A further analysis was conducted between the 2 extreme groups: extreme nonplayers (subgroup “never”) and extreme players (subgroups “several times a day” and “daily”), using nonparametric tests, such as the Mann-Whitney *U* test for independent samples. The statistical significance threshold was set at *P*<.05.

## Results

### Participant Flow

Ninety-two second year dental students were included in this study (mean age 19.49, SD 0.8 years; median age 20, IQR 19-21 years). Most of the students were women (n=66, 72%). Group allocation was based on responses to the question “How often do you think you play video games?” Thirty-seven participants were placed in the player group (40%), while 55 were placed in the nonplayer group (60%).

### Analysis of Outcome Measurements

There was no statistical difference between the player and nonplayer groups on the first test, assessing visuospatial ability (*P*=.051), and the modified PMD Test (*P*=.17), even if the nonplayer group tended to make slightly fewer mistakes on the first test and obtained a higher score on the second one. There was still no statistical difference on the third test (*P*=.87), but conversely, the nonplayer group tended to require slightly more time to perform the pulpotomy compared with the player group (Table 1).

**Table 1:** Comparison of scores on the 3 tests between the player and nonplayer groups.

	Nonplayers (n=55), mean (SD)	Players (n=37), mean (SD)	*P* value
Anatomy test, number of mistakes (out of 9)	2.75 (2.12)	3.67 (2.02)	.051
Modified Precision Manual Dexterity Test, score (out of 240)	146.65 (23.4)	139.36 (20.66)	.17
Pulpotomy test, time (in seconds)	311.85 (137.3)	306.92 (148.34)	.87

In addition, a subgroup analysis between the extremes of each group showed that the extreme nonplayer group appeared to make significantly fewer mistakes on the recognition test than the extreme player group (*P*=.048). However, there was no statistical difference between the extreme nonplayer group on the modified PMD Test (*P*=.22) or in the time required to perform the pulpotomy (*P*>.99) compared to the extreme player group (Table 2).

**Table 2:** Comparison of scores on the 3 tests between the extreme player and extreme nonplayer groups.

	Extreme nonplayers (n=34), mean (SD)	Extreme players (n=9), mean (SD)	*P* value
Anatomy test, number of mistakes (out of 9)	2.5 (2.09)	4 (1.87)	.048
Modified Precision Manual Dexterity Test, score (out of 240)	147.09 (24.34)	138.67(16.23)	.22
Pulpotomy test, time (in seconds)	296.7 (123.32)	321.56 (190.38)	>.99

## Discussion

### Principal Findings

The potential of video games as a tool to enhance manual dexterity and cognitive skills has been a subject of considerable interest. This study attempted to explore whether this potential may extend to the field of dentistry.

The first test was conceived with the objective of appraising the visuospatial aptitudes of the participants. In contrast to our findings, Mallow et al [22] demonstrated that participants who were novices in bronchoscopy performed better on a mental rotation test of a 3D figure than nonplayers prior to any training. To measure this, the authors used a psychometric test (the Purdue Spatial Visualization Test), which revealed a significant discrepancy between the 2 groups with respect to their baseline performance. In contrast, Kennedy et al [23] did not demonstrate a substantial discrepancy in mental capacity for 3D representations between players and nonplayers.

The second test assessed subjects’ psychomotor skills with a modified PMD Test. Kennedy et al [23] also showed that students who played video games at least 7 hours per week demonstrated significantly better psychomotor skills than students who did not play video games regularly, suggesting that video gaming experience may facilitate surgical learning [23]. According to Rosser et al [15], students who were players made fewer mistakes than nonplayers. In addition, if they played more than 3 hours per week, their performance increased. Sammut et al [14] observed 3 surgical tasks performed by young physicians and evaluated them. The results were correlated with the physicians’ previous experience with video games; the researchers found that the physicians who had played video games had a better accuracy rate.

The third test aimed to evaluate the speed of performing a technical procedure. Contrary to our results, Dye et al [7] suggested that action video games could significantly reduce reaction time without compromising accuracy. Moreover, according to Rosser et al [15], player participants executed a task more quickly than nonplayers. Shane et al [5] also demonstrated that students with video game experience had a shorter learning curve than those without, especially when learning 2 concurrent tasks. Without any medical knowledge, and with only a few instructions on how to perform the task, adolescent players were faster than experienced surgeons on simulators, emphasizing the importance of the ease with which these tools can be used [24]. Thus, despite a lack of medical knowledge, handling the training tools was easier for the adolescents than for the experienced surgeons.

A lot of effort has been devoted to quantify the impact of game-based training on cognitive abilities, and a significant proportion of research has been dedicated to exploring the formative role of video gaming and its influence on the learning process [22,25]. As hypothesized, video games may serve as a complementary training tool in the acquisition of surgical skills; this is supported by their historical use in simulation-based training [17]. Moreover, the transfer effect increases as visual similarity increases [26]. Thus, performance in spatially intense and visually challenging video games could be a predictor of surgical simulation outcomes. Past studies have also highlighted the importance of video gaming typology in participants’ previous experience [27,28]. Players differ according to their habits, such as the type of games they play or the frequency with which they play them. Indeed, as has been demonstrated in previous studies, players of action games appear to demonstrate superior visuomotor control and faster information processing speeds than players of nonaction games [27,29]. Indeed, surgical performance criteria were enhanced most in players of action games. It is posited that action games help reduce the processing time of information requiring a rapid response, without altering the motor response [30]. However, most studies do not clearly distinguish between gaming experience and gaming skill, which remain poorly defined concepts [14].

Harrington et al [31] investigated the relationship between visuospatial performance and nonsurgical experiences during the learning process. Participants were assessed during the skill acquisition phase and the maintenance phase. Players were found to perform better than other participants during the maintenance phase. Middleton et al [32] suggested that for educational purposes, practicing video games could improve bimanual dexterity, help train the surgeon’s nondominant hand, and accelerate the acquisition of basic surgical skills. Psychologically, this enhanced performance could relate to the “flow” concept described by Csikszentmihalyi, an immersive mental state of deep concentration and satisfaction [33]. This contributes to the rationale for active and immersive learning strategies. Nevertheless, contradictory results also exist [18,34], preventing definitive conclusions. In several studies, performance improvements in players appeared only after repeated attempts, suggesting a potentially accelerated learning curve rather than superior baseline skills.

### Limitations

Several methodological limitations of this study should be considered. There were unequal sample sizes between groups, with the player group being smaller. This may have reduced the statistical power and precision of the results. As this was the first study on this topic in the field of dental education, we drew comparisons with research on medical students at more advanced levels, limiting generalizability. Discipline-specific testing tools were used, which may not be valid in the context of video game–related research, hindering comparisons with the existing literature. Furthermore, no restrictions were placed on game types or platforms—given that different genres develop different skills, this may have obscured specific effects of action-heavy games. Self-reported gaming frequency may also lack accuracy, and categorical ranges (eg, “occasionally”) were not precisely standardized.

Improvements should be considered for future studies, such as a longitudinal design, which would allow evaluation of learning progression over time [31,35]. This preliminary work has facilitated the validation of 2 of the 3 measurement tools for such a longitudinal study, which could aim to observe the learning curve for technical skills among dental students in relation to their video game–playing habits. Indeed, the simulation of technical skills using a pulpotomy test and the modified PMD Test appear to be relevant measurement tools. However, the visuospatial aptitude test does not appear to be an appropriate instrument for the population under study. As this was a pilot study, we tried to create a tool that would evaluate the concept of visuospatial assessment but be specific to the field of dentistry and easy for students to use and understand. However, the lack of scope (the total score was only 9) and the difficulty students had in understanding it during assessment sessions, as well as its lack of validation, lead us to question its future use. Vandenberg and Kuse [36] introduced an alternative method, the mental rotation test, which aims to assess these skills prior to the start of studies. Collecting participants’ feedback on task difficulty could identify barriers and refine test calibration. Nevertheless, this preliminary study allowed identification of key methodological issues ahead of the now-published protocol for a prospective longitudinal investigation [37].

### Conclusion

The results of this preliminary study did not demonstrate that regular video game playing improved basic preclinical performance in novice dental students. Nevertheless, this work enabled the refinement and validation of assessment instruments for further research. Future longitudinal studies are necessary to determine whether dental students who engage in gaming acquire technical skills more efficiently during preclinical training than students who do not.

## Abbreviations

PMD Test: Precision Manual Dexterity Test

## Data Availability

The data sets generated during and/or analyzed during this study are available from the corresponding author on reasonable request.
